# Importance of competing risks in the analysis of anti-epileptic drug failure

**DOI:** 10.1186/1745-6215-8-12

**Published:** 2007-03-29

**Authors:** Paula R Williamson, Catrin Tudur Smith, Josemir W Sander, Anthony G Marson

**Affiliations:** 1 Centre for Medical Statistics and Health Evaluation, University of Liverpool, Liverpool, UK; 2 Institute of Neurology, University College London, London, UK; 3 Division of Neurological Sciences, University of Liverpool, Liverpool, UK

## Abstract

**Background:**

Retention time (time to treatment failure) is a commonly used outcome in antiepileptic drug (AED) studies.

**Methods:**

Two datasets are used to demonstrate the issues in a competing risks analysis of AEDs. First, data collection and follow-up considerations are discussed with reference to information from 15 monotherapy trials. Recommendations for improved data collection and cumulative incidence analysis are then illustrated using the SANAD trial dataset. The results are compared to the more common approach using standard survival analysis methods.

**Results:**

A non-significant difference in overall treatment failure time between gabapentin and topiramate (logrank test statistic = 0.01, 1 degree of freedom, p-value = 0.91) masked highly significant differences in opposite directions with gabapentin resulting in fewer withdrawals due to side effects (Gray's test statistic = 11.60, 1 degree of freedom, p = 0.0007) but more due to poor seizure control (Gray's test statistic = 14.47, 1 degree of freedom, p-value = 0.0001). The significant difference in overall treatment failure time between lamotrigine and carbamazepine (logrank test statistic = 5.6, 1 degree of freedom, p-value = 0.018) was due entirely to a significant benefit of lamotrigine in terms of side effects (Gray's test statistic = 10.27, 1 degree of freedom, p = 0.001).

**Conclusion:**

Treatment failure time can be measured reliably but care is needed to collect sufficient information on reasons for drug withdrawal to allow a competing risks analysis. Important differences between the profiles of AEDs may be missed unless appropriate statistical methods are used to fully investigate treatment failure time. Cumulative incidence analysis allows comparison of the probability of failure between two AEDs and is likely to be a more powerful approach than logrank analysis for most comparisons of standard and new anti-epileptic drugs.

## Background

Patients with a new diagnosis of epilepsy are usually prescribed an antiepileptic drug (AED) as monotherapy [1,2]. An AED would be considered successful if the person taking it becomes seizure free with few side effects. Treatments that cause unacceptable side effects are likely to be changed to an alternative, whilst a treatment that fails to control seizures will either be changed to an alternative, or a second drug will be added. In order to assess this measure of failure in randomised trials (RCTs) of AEDs, a primary outcome recommended by the International League Against Epilepsy is retention time on AED [3], a composite outcome [4,5], defined as the time from randomisation to the withdrawal of the randomised AED or addition of another.

Overall retention on treatment, i.e. time to treatment failure for any reason, may be analysed using standard survival analysis methods [6] and such analyses are common in the AED field in both studies of monotherapy [7-14] and add-on therapy [15,16]. However this approach fails to examine different reasons for treatment failure (such as inadequate seizure control or adverse events), and assumes that reasons for failure are of equal importance which may not be the case. For example the loss of a driving license due to continued seizures will have differing social and economic consequences to the more common side effects such as nausea, dizziness or rash. A situation could arise in which two AEDs are considered equivalent as a result of similar overall treatment failure when in fact the drugs have very different effects on withdrawal due to side effects and poor seizure control.

In the statistical literature, the situation where there are several reasons why an event can occur is known as 'competing risks'. Ignoring this aspect of an outcome by analysing events overall can result in misleading conclusions [6]. Some authors have examined separately the time to withdrawal due to side effects [16] and have censored patients whose allocated treatment is changed due to inadequate seizure control, which may give misleading results as analyses assume that the competing risks of withdrawal are independent [6]. This assumption is questionable for AED treatment where there may be an association between these two causes of treatment failure. Thus a full investigation of retention time should include statistical methods that do not assume the competing risks of withdrawal are independent.

This paper considers the implications of competing risks of treatment failure for the design, data collection and analysis of AED studies motivated by analysis of the SANAD (Standard And New Anti-epileptic Drugs) trial [17] and drawing on our experience of datasets from our programme of individual patient data meta-analyses (IPDMA). We make recommendations for improving research practice.

## Methods

### Treatment failure time

The definition of treatment failure time used in this paper is the time from randomisation to the intention to withdraw the randomised drug due to lack of efficacy (poor seizure control) and/or intolerable side-effects; or the addition of other AEDs whichever is the earliest. The date of intention to withdraw rather than completion of withdrawal was chosen to reflect the point at which the treatment policy had been changed. Although retention time is the usual term for this outcome, a more appropriate alternative is time to treatment failure since it better reflects the event of interest. It also encompasses the situation when an AED is added as a result of poor seizure control and not just treatment failures resulting in the withdrawal of the original drug. Patients who achieve a period of seizure freedom (usually 2 to 3 years) may decide to withdraw treatment, in this circumstance the withdrawal reflects a successful outcome (seizure remission) rather than a treatment failure and the withdrawal is not counted as an event. Since remissions occur later in the follow-up period than withdrawals due to treatment failure, such censoring has no effect on the analysis.

### Participants

Two datasets are used to demonstrate the issues in a competing risks analysis of AEDs. First, data collection, terminology and follow-up considerations are discussed with reference to information from a monotherapy meta-analysis. Recommendations for improved data collection and analysis methods are then illustrated using the SANAD dataset.

### Individual patient data meta-analysis dataset

Information on treatment failure time was available from 15 randomised trials [18-31] involving 3883 individuals, collected as part of a suite of individual patient data meta-analyses concerning six different AEDs [7-14].

### SANAD dataset

The design and analysis of this trial have been published elsewhere [17]. For illustration here, we present the results from a competing risks analysis of the data for two pairwise comparisons, (i) gabapentin (GBP) versus topiramate (TPM) and (ii) lamotrigine (LTG) versus carbamazepine (CBZ).

### Design issues

Table 1 summarises the information collected on reason for treatment failure in the 15 trials where we had obtained the individual patient data. There was no standard approach for collecting this information and the level of detail recorded varied across the studies. For this reason, no analysis of competing risks has been undertaken for these data in the relevant meta-analyses. In some trials, only the primary reason for AED withdrawal was recorded. In others, several reasons were recorded along with order of importance. Occasionally, a further variable was recorded which indicated whether the clinician considered the reason for withdrawal to be related to the AED or not.

**Table 1:** Classification of reasons for treatment change in 15 monotherapy trials

**Trial**	**Still on drug at end of follow-up (censored)**	**Reason for withdrawal**					
		
		**Adverse events**	**Seizure control**	**Remission**	**Seizure control and adverse events**	**Unclear**	**Total number of patients in trial**
Heller et al., 1995^18^	131	25	29	36	15	7	243
De Silva et al., 1996^19^	41	9	35	59	19	4	167
Mattson et al., 1985^20^	312	25	16	0	108	14	475
Mattson et al., 1992^21^	291	68	50	0	0	71	480
Richens et al., 1994^22^	196	37	34	0	2	23	292
Verity et al., 1995^23^	175	23	30	0	11	7	246
Brodie et al., 1995a^24^	85	40	3	0	2	6	136
Brodie et al., 1995b^24^	90	28	4	0	0	2	124
Ramsay et al., 1992^25^	112	10	3	0	0	11	136
Turnbull et al., 1985^26^	97	5	20	0	0	18	140
Placencia et al., 1993^27^	159	10	0	0	0	23	192
Brodie et al., 1999^28^	106	40	2	0	2	0	150
Bill et al., 1997^29^	239	22	2	0	0	24	287
Guerreiro et al., 1997^30^	158	16	7	0	1	11	193
Nieto-Barrera et al., 2001^31^	534	70	18	0	0	0	622

In some trials patients may have been recorded as having withdrawn for both side effects and poor seizure control. In clinical practice, if a patient is still experiencing seizures the dose of medication is increased until either seizures are controlled or side effects occur that prevent further dose increases. In the latter case, treatment will be withdrawn because of both inadequate seizure control and side effects. Since the former is the primary reason for failure, patients are classified as failures due to inadequate seizure control. Some patients will reach maximum doses without control of seizures and without significant side effects. Pragmatically, the clinician would call this inadequate seizure control.

Table 1 shows that the percentage of patients for whom the reason for treatment failure was unclear varied from 0 to 15%, but as a proportion of the number of withdrawals ranged as high as 70%. Recording the reason for AED withdrawal as non-compliance is not sufficient. Non-compliance may be as a result of side effects or possibly poor seizure control. Alternatively it may be due to a reason unrelated to the efficacy or tolerability of the drug. Thus to classify correctly the information in both an overall treatment failure analysis and a competing risks analysis requires more information to be obtained.

As a result of the problems identified in data collection in previous trials, extra detail regarding treatment failure reason was sought in the SANAD study. Table 2 shows the categories for reason for withdrawal recorded in SANAD, together with whether the withdrawal was coded as an event, and if so the reason for withdrawal (ISC or UAE), or as a censored observation in the competing risks analysis of treatment failure time.

**Table 2:** Classification of reasons for treatment change used in the SANAD trial

**Reason for withdrawal from drug or other drug added (earliest event)**	**Categorised as event (type classified as ISC or UAE) or censored in 'time to treatment failure' analysis**	**Number of patients (CBZ, LTG, TPM and GBP)**
Inadequate seizure control	Event (ISC)	348
Unacceptable adverse events	Event (UAE)	320
Perceived risk of adverse effect	Event (UAE)	8
Remission of epilepsy categorised by patient^**1 **^(less than 12 months' remission from seizures)	Event (UAE)	46
Remission of epilepsy categorised by clinician (any length) or patient (more than 12 months' remission from seizures)	Censored	88
Study withdrawal – consent withdrawn^**2**^	Censored	23
Death (unrelated to epilepsy)^**3**^	Censored	32
Death (related to epilepsy)^**3**^	Event (ISC)	3
Moved from area	Censored	0
Patient non-compliant or patient decision^**4**^	Censored	12
Pregnant or planning pregnancy	Censored	6
Other	Censored	12
Still on drug at last follow-up	Censored	597
Not epilepsy	Excluded	16

^**1 **^Patients decision to withdraw before 12 months' freedom from seizures is likely to be highly influenced by side effects or perception of side effects

^**2 **^Study withdrawals are automatically checked to ensure that the patient wants to withdraw from study rather than from drug only

^**3 **^Blinded assessment to identify whether death is related or unrelated to epilepsy/AED to take place prior to analysis

^**4 **^Further information is sought if patient withdraws from drug due to "non-compliance" as the underlying reason could be unacceptable adverse events, inadequate seizure control or remission of epilepsy.

### Statistical analysis

Standard survival analysis methods such as Kaplan-Meier survival curve estimation, logrank hypothesis testing and Cox regression modelling can all be applied validly to the analysis of overall treatment failure time. However Kaplan-Meier estimates of being event free for a specific cause will be biased if the assumption that the competing risks are independent is violated. Kaplan-Meier curves are presented in this paper for illustration only however they cannot be interpreted in terms of survival probabilities in the presence of dependent censoring. We describe an alternative approach, cumulative incidence analysis, which makes no such assumption and allows the assessment of cause-specific withdrawal in the presence of other competing risks in the Appendix.

If all events of one type occur after the last event of another type, the standard survival analysis methods such as the logrank test and the Cox model will give identical results to those obtained following the cumulative incidence approach. Thus as a first stage of data analysis, it is important to understand the time distribution of the various causes for withdrawal.

Cumulative incidence analysis, including hypothesis testing, is available as part of the 'cmprsk' module [32] within the R software package. Version 2.1–5 was used for the following analyses.

## Results

**Figure 1:**
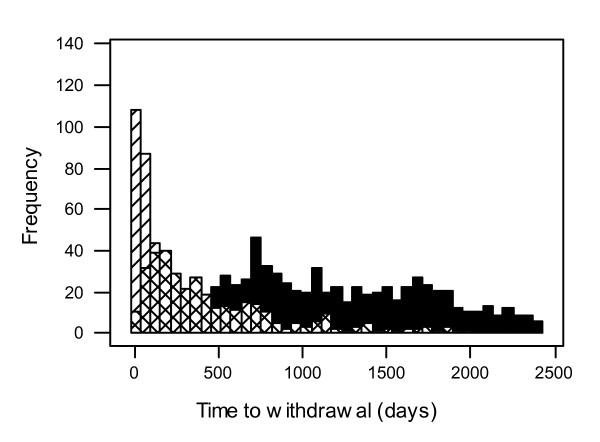
**Distribution of withdrawal times for various reasons in the SANAD trial**. Censored denoted by full shading, Unacceptable Adverse Event by /, Inadequate Seizure Control by X.

Figure 1 shows how the distributions of withdrawal times due to side effects and poor seizure control in the SANAD trial overlap thus indicating the need to consider a cumulative incidence approach.

**Figure 2:**
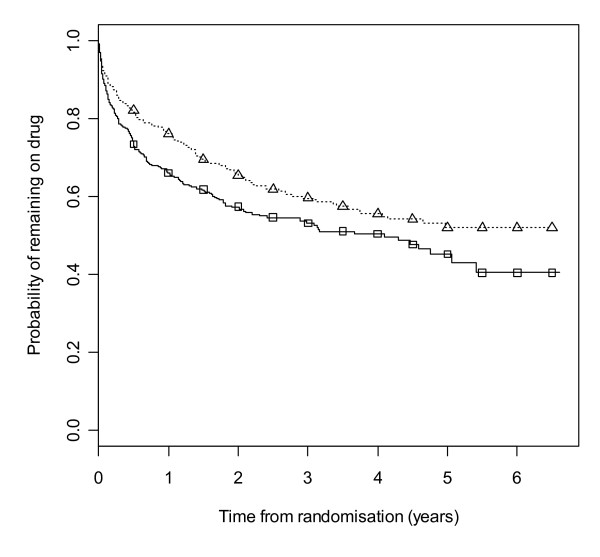
**Kaplan-Meier distribution of overall treatment failure time for carbamazepine versus lamotrigine**. Lamotrigine group denoted by triangle, carbamazepine by square.

**Figure 3:**
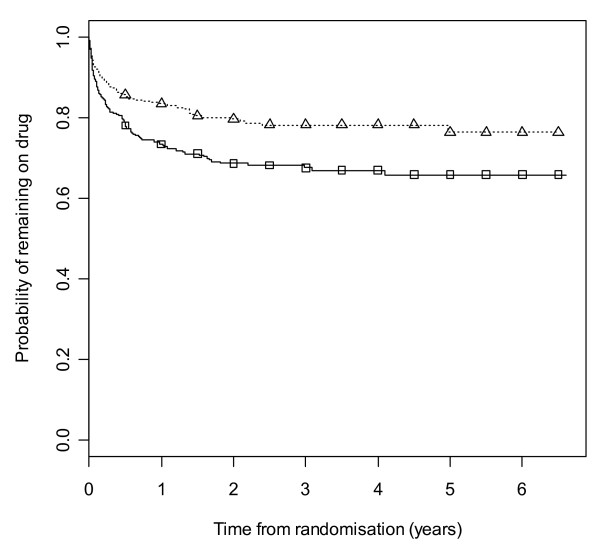
**Kaplan-Meier distribution of time to withdrawal due to side effects for carbamazepine versus lamotrigine**. Lamotrigine group denoted by triangle, carbamazepine by square.

**Figure 4:**
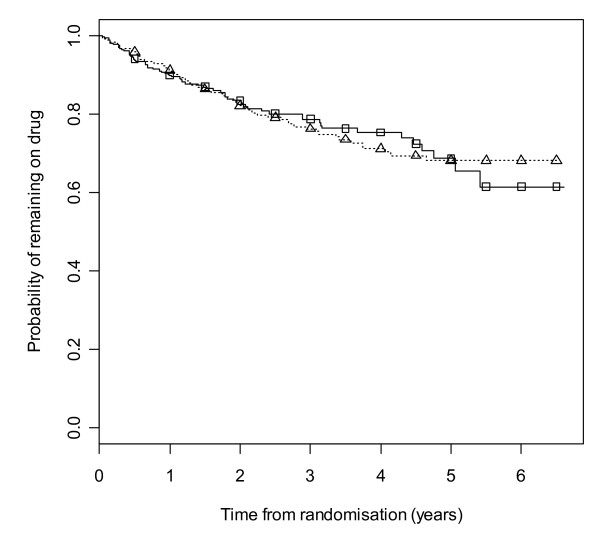
**Kaplan-Meier distribution of time to withdrawal due to poor seizure control for carbamazepine versus lamotrigine**. Lamotrigine group denoted by triangle, carbamazepine by square.

Figure 2 gives results for overall treatment failure for CBZ and LTG when the two causes, side effects and poor seizure control, are not distinguished as events. The logrank test statistic is 5.6 (1 df, p-value = 0.018) indicating LTG is better retained than CBZ. Figure 3 considers withdrawal for side effects as the only event, but censors withdrawals for poor seizure control at the time of withdrawal. The logrank test statistic is 10.4 (1 df, p-value = 0.001). There is clear evidence that LTG is better tolerated than CBZ with differences emerging very early on. Figure 4 considers withdrawal for poor seizure control as the only event, but censors withdrawals for side effects at the time of withdrawal. The logrank test statistic is 0.02 (1 df, p-value = 0.89) and shows LTG to be equivalent to CBZ in terms of seizure control.

**Figure 5:**
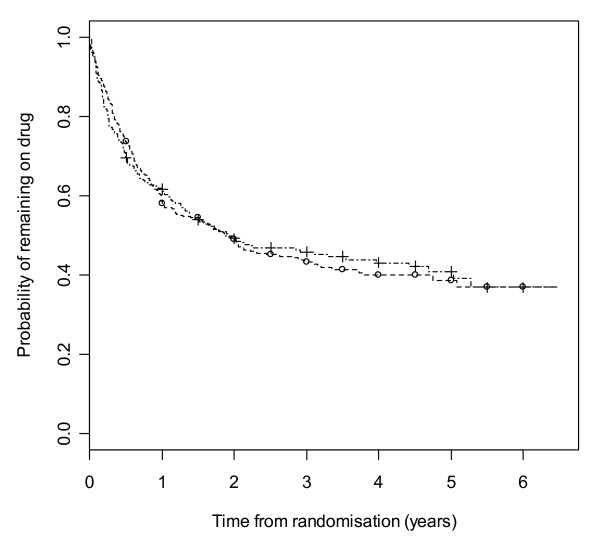
**Kaplan-Meier distribution of overall treatment failure time for gabapentin versus topiramate**. Gabapentin group denoted by circle, topiramate by line.

**Figure 6:**
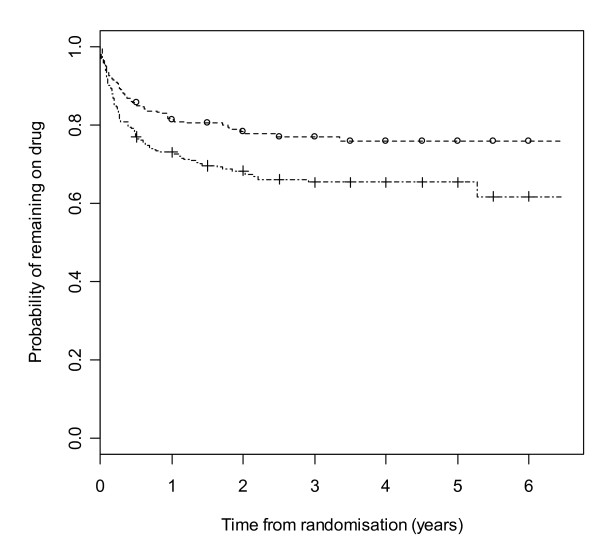
**Kaplan-Meier distribution of time to withdrawal due to side effects for gabapentin versus topiramate**. Gabapentin group denoted by circle, topiramate by line.

**Figure 7:**
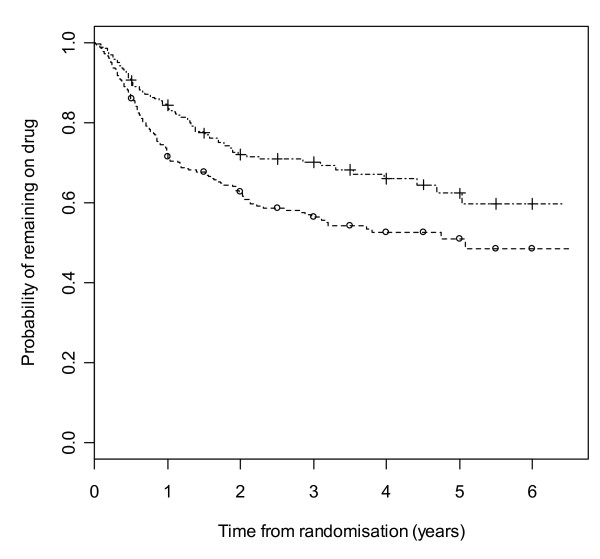
**Kaplan-Meier distribution of time to withdrawal due to poor seizure control for gabapentin versus topiramate**. Gabapentin group denoted by circle, topiramate by line.

Figure 5 gives results for overall treatment failure for GBP and TPM. The logrank test statistic is 0.01 (1 df, p-value = 0.91) indicating no overall difference between GBP and TPM. Figure 6 considers withdrawal for side effects as the only event, but censors withdrawals for poor seizure control at the time of withdrawal. The logrank test statistic is 9.6 (1 df, p-value = 0.002). There is clear evidence that GBP is better tolerated than TPM with differences emerging very early on. Figure 7 considers withdrawal for poor seizure control as the only event, but censors withdrawals for side effects at the time of withdrawal. The logrank test statistic is 9.3 (1 df, p-value = 0.002). There is clear evidence that TPM improves seizure control compared to GBP with differences emerging very early on.

**Figure 8:**
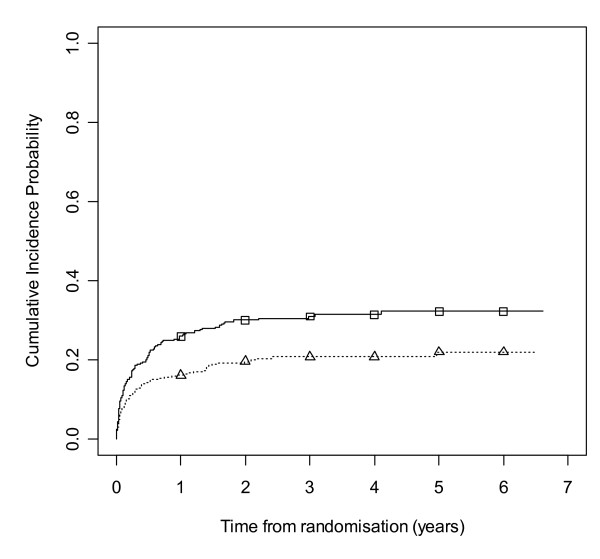
**Cumulative incidence curves for withdrawal due to side effects for carbamazepine versus lamotrigine**. Lamotrigine group denoted by triangle, carbamazepine by square.

**Figure 9:**
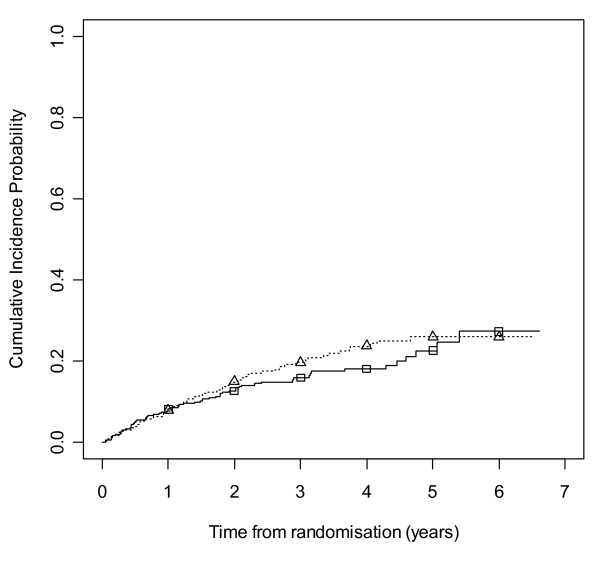
**Cumulative incidence curves for withdrawal due to poor seizure control for carbamazepine versus lamotrigine**. Lamotrigine group denoted by triangle, carbamazepine by square.

Figures 8 and 9 show cumulative incidence plots for the two competing risks. The test statistic [32] comparing withdrawals due to side effects is 10.27 (1 df, p-value = 0.001). The test statistic comparing withdrawals due to poor seizure control is 0.78 (1 df, p-value = 0.38). The plots show the probabilities of suffering a treatment failure due to an adverse event and of suffering a failure due to inadequate seizure control in the setting where competing risks are acknowledged to exist. Thus at two years, for LTG the chance of withdrawal due to adverse events is 20% and of withdrawal due to inadequate seizure control is 15%, whilst for CBZ these probabilities are 30% and 13% respectively.

**Figure 10:**
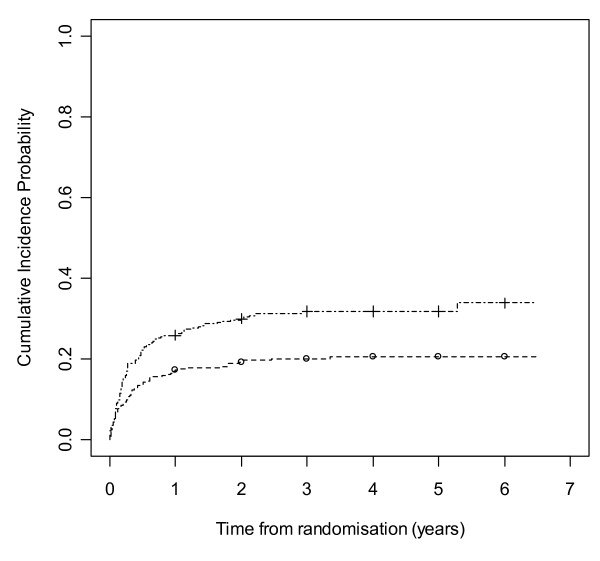
**Cumulative incidence curves for withdrawal due to side effects for gabapentin versus topiramate**. Gabapentin group denoted by circle, topiramate by line.

**Figure 11:**
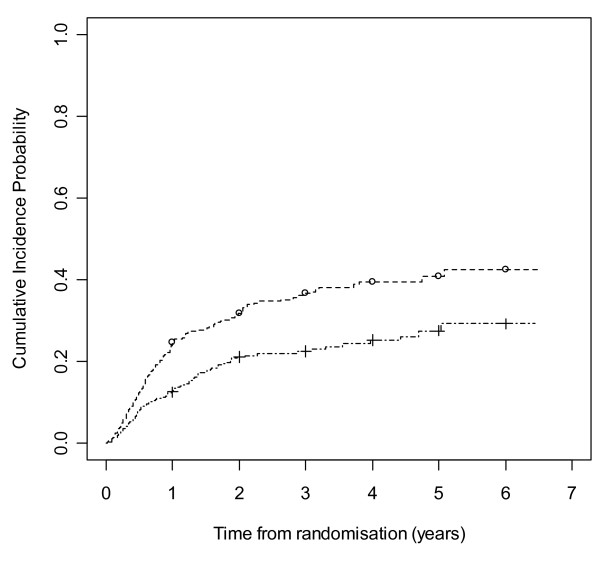
**Cumulative incidence curves for withdrawal due to poor seizure control for gabapentin versus topiramate**. Gabapentin group denoted by circle, topiramate by line.

Figures 10 and 11 show cumulative incidence plots for the two competing risks. The test statistic [32] comparing withdrawals due to side effects is 11.60 (1 df, p-value = 0.0007). The test statistic comparing withdrawals due to poor seizure control is 14.47 (1 df, p-value = 0.0001). The cumulative incidence analysis provides stronger evidence of the advantage to GBP for side effects and TPM for seizure control compared to the logrank approach. The plots show the probabilities of suffering a treatment failure due to an adverse event and of suffering a failure due to inadequate seizure control in the setting where competing risks are acknowledged to exist. Thus at two years, for GBP the chance of withdrawal due to adverse events is 19% and of withdrawal due to inadequate seizure control is 32%, whilst for TPM these probabilities are 30% and 21% respectively.

## Discussion

Failure to investigate the competing risks of anti-epileptic drug withdrawal can lead to important differences in the clinical effect profiles of AEDs being missed. Thus overall treatment failure could be similar for two AEDs when the drugs have very different effects on withdrawal due to side effects and poor seizure control. For a particular patient, it is best to summarise each of these two risks for AEDs individually, as well as considering the overall risk of withdrawing for any reason. Further research is needed however to ascertain the relative importance of these different outcomes to individuals with epilepsy.

Prior to analysis, the timescale for competing causes should always be investigated. Risks operating over different time periods are less of an issue for the analysis. However, as demonstrated here, there is overlap of the timescale for withdrawals due to the different reasons and this is likely to be true for all AED studies.

More attention is required in the choice of statistical methods employed for the analysis of the competing risks of drug withdrawal. Cumulative incidence estimation allows appropriate inference about the probability of failure of AEDs allowing for the presence of competing reasons for such failure. This contrasts with the inappropriate inference that is often made from one minus the Kaplan-Meier estimate for a single failure reason which can only be interpreted as a probability of failure in the hypothetical situation that other failure reasons are not possible. Further, as suggested by a recent simulation study [33], the cumulative incidence approach has greater power to detect treatment differences than a logrank analysis in particular circumstances. This holds true for a difference in one direction for one cause and no or an opposite difference for the other cause, a situation worthy of note for anti-epileptic drug trials particularly when new and standard AEDs are being compared. Although conclusions from the two methods of analysis were similar for both pairwise comparisons shown here, this will not always be the case.

The reliability of classification of the cause of drug failure must be assured before a competing risks analysis is undertaken. The analysis of overall withdrawal is safe since the information that a drug has been withdrawn is usually reliable. For a competing risks analysis however, the analyst must consider whether the classification of the reason for withdrawal is reliable. The trials reviewed in this paper varied in the level of detail collected. In particular, data on the reason for withdrawal needs to be as accurate as possible for more explanatory research questions such as those posed in pharmacogenetic studies.

The dataset used for illustration here contained only two patients for whom the reason for drug withdrawal was recorded as non-compliance. Censoring the outcome for these two individuals implicitly assumes that the reason for non-compliance was not related to either seizure control or side effects. The results are likely to be robust to such an assumption in this example due to the low number of cases involved. However in other datasets where this reason is recorded more frequently, some sort of sensitivity analysis would be required to establish the robustness of the conclusions to this assumption. One approach would be to first code the withdrawal due to non-compliance as an event then as a censored observation and assess the robustness of the conclusions to such extreme assumptions. Of course ideally, as is the thrust of this paper, one would wish to minimise such problems through improved data collection methods.

## Recommendations

The work presented here leads to the following recommendations for future studies which include treatment failure time as an outcome:

1) the outcome should be defined as the time to the intention to withdraw the randomised AED or add in another (i.e. the point at which the treatment policy has been changed),

2) sufficient detail should be collected on the primary reason for drug withdrawal/addition to allow classification into one of the following categories: withdrawal due to unacceptable side effects, withdrawal due to inadequate seizure control, withdrawal due to remission, withdrawal due to a reason confirmed to be unrelated to either side effects or seizure control.

The majority of drug withdrawals occurred by one year in the SANAD dataset although two-year follow-up provides greater power since almost all drug withdrawals occurred by this time. Monotherapy studies need longer follow-up however to investigate seizure control. Studies with both treatment failure and remission outcomes should intend to follow up each patient for a minimum of one year, and ensure that a reasonable number of patients will provide longer term follow-up, particularly important for seizure outcomes. Finally, we recommend that this outcome be termed 'time to treatment failure' rather than retention time since the former better reflects the event of interest.

## Authors' contributions

PRW identified the issue of competing risks in epilepsy trials, investigated appropriate analysis methods, directed the analyses, interpreted results and wrote the manuscript. CTS carried out the statistical analyses, interpreted results and contributed to writing the manuscript. JWS provided data for analysis, provided clinical input and commented on the manuscript. AGM provided data for analysis, provided clinical input and commented on the manuscript.

## Appendix

The non-parametric cause-specific hazard function for cause *l *is estimated by maximum likelihood via

hl(tj)=dljnj
 MathType@MTEF@5@5@+=feaafiart1ev1aaatCvAUfKttLearuWrP9MDH5MBPbIqV92AaeXatLxBI9gBaebbnrfifHhDYfgasaacH8akY=wiFfYdH8Gipec8Eeeu0xXdbba9frFj0=OqFfea0dXdd9vqai=hGuQ8kuc9pgc9s8qqaq=dirpe0xb9q8qiLsFr0=vr0=vr0dc8meaabaqaciaacaGaaeqabaqabeGadaaakeaacqWGObaAdaWgaaWcbaGaemiBaWgabeaakiabcIcaOiabdsha0naaBaaaleaacqWGQbGAaeqaaOGaeiykaKIaeyypa0ZaaSaaaeaacqWGKbazdaWgaaWcbaGaemiBaWMaemOAaOgabeaaaOqaaiabd6gaUnaaBaaaleaacqWGQbGAaeqaaaaaaaa@3C9B@

where *d*_*lj *_represents the number of failures of type *l *at time *t*_*j *_and *n*_*j *_is the number at risk at this time. The maximum likelihood estimator of the cause-specific incidence at time *t*_*j *_is

*I*_*l*_(*t*_*j*_) = *P*(*T *<*t*_*j*_, *L *= *l*) = *S*(*t*_*j*-1_)*h*_*l*_(*t*_*j*_)

where *P*(*T *<*t*_*j*_, *L *= *l*) is the probability that an individual withdraws from drug due to cause *l *before *t*_*j *_and *S*(*t*_*j*-1_) denotes the overall survival function at time *t*_*j*-1 _i.e. the probability that an individual does not withdraw for any reason before *t*_*j*-1_.

The cause-specific cumulative incidence function at time t is then

Il(t)=P(T<t,L=l)=∑j=1rIl(tj)
 MathType@MTEF@5@5@+=feaafiart1ev1aaatCvAUfKttLearuWrP9MDH5MBPbIqV92AaeXatLxBI9gBaebbnrfifHhDYfgasaacH8akY=wiFfYdH8Gipec8Eeeu0xXdbba9frFj0=OqFfea0dXdd9vqai=hGuQ8kuc9pgc9s8qqaq=dirpe0xb9q8qiLsFr0=vr0=vr0dc8meaabaqaciaacaGaaeqabaqabeGadaaakeaacqWGjbqsdaWgaaWcbaGaemiBaWgabeaakiabcIcaOiabdsha0jabcMcaPiabg2da9iabdcfaqjabcIcaOiabdsfaujabgYda8iabdsha0jabcYcaSiabdYeamjabg2da9iabdYgaSjabcMcaPiabg2da9maaqahabaGaemysaK0aaSbaaSqaaiabdYgaSbqabaGccqGGOaakcqWG0baDdaWgaaWcbaGaemOAaOgabeaakiabcMcaPaWcbaGaemOAaOMaeyypa0JaeGymaedabaGaemOCaihaniabggHiLdaaaa@4DE0@

where the summation is over each cause-specific event time up to time t.
